# Radiologic and Intraoperative Finding of Intimal Tear in Type A Intramural Hematoma of the Aorta

**DOI:** 10.1055/s-0039-1678569

**Published:** 2019-02-22

**Authors:** Gianfranco Filippone, Giovanni Caruana, Violetta Moscaritolo, Sara Rita Vacirca, Vincenzo Argano

**Affiliations:** 1 Unit of Cardiac Surgery, University of Palermo, Palermo, Italy; 2 Department of Radiology, University of Palermo, Palermo, Italy; 3 Intensive Care Unit, University of Palermo, Palermo, Italy; 4 Unit of Cardiac Surgery, University of Roma Tor Vergata, Rome, Italy

**Keywords:** intramural hematoma, aortic dissection, intimal tear

## Abstract

Aortic intramural hematoma (IMH) is described as “dissection without intimal tear” due to rhexis of vasa vasorum, which results in bleeding within the tunica media in the absence of intimal disruption or blood flow communication. The aim of our study is to validate perioperative evidence of intimal entry tear in IMH patients and to suggest that this entity may represent a part of a disease and not a separate disease.

A 78-year-old woman, hypertensive, was admitted to our hospital for acute chest pain. An unenhanced computed tomography (CT) scan was performed, which showed an aneurysm of the thoracic aorta (6 cm) complicated by Type A intramural hematoma (IMH) ( Fig. 1A , B ). After contrast medium administration, a localized blood-filled pouch protruding from the true lumen into the thrombosed false lumen of the aorta was also detected in the ascending aorta, 1 cm before the origin of brachiocephalic artery ( Fig. 1C – E ; Video 1 ), configuring a so-called ulcer-like projection. The patient was referred for surgery. During hypothermic circulatory arrest, the hematoma ( Fig. 2A ) was evacuated. A linear-shaped intimal tear, measuring 1.5 cm, was identified 1 cm before the origin of brachiocephalic artery ( Fig. 2B ). A hemiarch procedure associated to root replacement with a biological valved conduit using two-graft technique was performed ( Fig. 2C ). The postoperative course was uneventful, and the patient was discharged home on the 13th postoperative day.

**Fig. 1:**
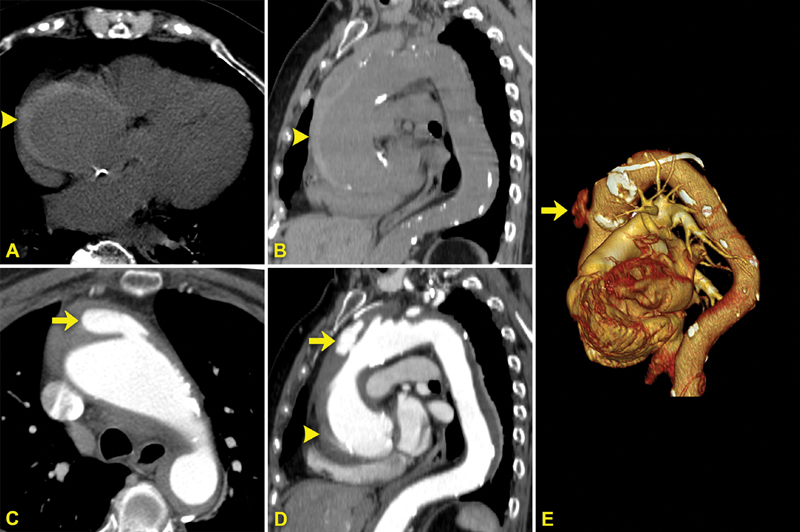
( **A** ) Axial and ( **B** ) sagittal unenhanced CT images show a crescent-shaped thickening of the aortic wall (arrowheads) with greater attenuation than the lumen, characteristic for intramural hematoma (IMH). ( **C** ) Axial and ( **D** ) sagittal contrast-enhanced CT images show a localized blood-filled pouch (arrows) protruding into the IMH from the aortic lumen through an intimal lesion, characteristic for ulcer-like projection (ULP). ( **E** ) Volume rendering reconstruction showing ULP in the anterior wall of the ascending aorta.

**Fig. 2:**
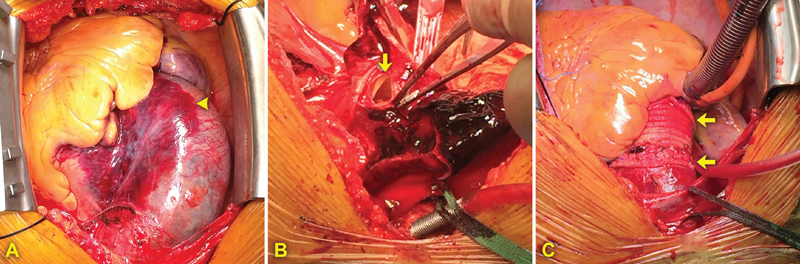
( **A** ) Intraoperative view of the IMH (arrowhead). ( **B** ) Intraoperative findings of 1.5 cm linear-shaped intimal tear (arrow). ( **C** ) Hemiarch + biological valved conduit repair procedure; arrows show distal and graft-to-graft anastomosis.

**Video 1**

Axial computed tomography animation showing Type A intramural hematoma (IMH) and ulcer-like projection (ULP) of the ascending aorta.

Since the first description, IMH was reported as a clinical entity defined as “dissection without intimal tear due to rhexis of vasa vasorum,” but this theory has not been scientifically validated. Some authors believe that all IMH cases are acute dissections with thrombosis of the false lumen and that an intimal tear is always present, but it cannot be identified; thus, IMH could not exist. 1 The newest improvements in noninvasive diagnostic imaging techniques, particularly multidetector CT, have recently permitted to identify lesions that could be considered as intimal tears. 2 3 The CT finding of an intimal lesion in the case, we present herein is intraoperatively validated, is in agreement with the aforementioned hypothesis, suggesting that IMH may represent a part of a disease (acute aortic dissection) and not a disease apart.
